# Bogus Allergy Treatment

**Published:** 1987-02

**Authors:** T. J. David

**Affiliations:** Senior Lecturer in Child Health, University of Manchester Honorary Consultant Paediatrician, Booth Hall Children's Hospital, Manchester


### Bogus Allergy Treatment

**Dr T. J. David, MB ChB (Bristol), PhD (Bristol), MD (Bristol), FRCP, DCH**
Senior Lecturer in Child Health, University of Manchester
Honorary Consultant Paediatrician, Booth Hall Children's Hospital, Manchester

### INTRODUCTION

The public have developed an increasing appetite for self-help, alternative medicine, and allergy. This has been associated with the development outside the National Health Service of self-referral laboratories and various allergy, ecology and environmental therapy clinics. Patients with chronic, intractable or incurable diseases are a vulnerable group, and anecdotal media reports of unlikely and unorthodox cures inevitably tempt some of the more gullible and desperate. Alternative medicine, clinical ecology and environmental therapy are being increasingly exploited commercially, and in turn in our Department we are spending an increasing amount of time trying to salvage the refugees from such treatment (1). Unfortunately, despite much research, there are still no valid laboratory tests to diagnose food allergy, leaving the way clear for quacks and charlatans to exploit anxious patients or parents. Many doctors are unaware of the depth of irrationality which is sometimes involved in unorthodox allergy treatment. The various forms of unorthodox or bogus allergy diagnosis and treatment in current use in this country are briefly described here.

### RADIONICS

Radionics, radiaesthesia, psionic medicine and dowsing are closely related practices and employ extra-sensory perception. The basis of radionic theory is the concept that "all life forms, including man, are submerged in and interpenetrated by a common field of energy. At the lowest level this field registers as the electromagnetic spectrum but there are many levels or planes of energy which cannot be measured by scientific instrumentation, and which lie beyond the electromagnetic field" (2). The patient does not need to be present for radionic diagnosis or treatment. This is due to the supposition that the practitioner and patient are connected by a beam of energy along which information can be derived relevant to the patient's health. By similar reasoning, "energy" for treatment purposes can be "broadcast" to the distant patient (2).

Radionic diagnosis usually employs a pendulum, also known as a dowser, although sometimes it uses a radionic box or instrument, which is claimed to "objectify a process going on at mental levels" (2). A piece of filter paper moistened with a drop of blood, saliva or urine, or a sample of hair can all serve as a "witness" of the patient. The pendulum is "susceptible to paranormal influences which will affect its mode of oscillation, and so provide a basis of communication with the problem under investigation" (3). The pendulum is used to "interrogate" a witness to obtain medical information. For those with special gifts, a pendulum may not be necessary at all, and one authority reported that he could dowse with his mind, "with a measure of clairaudience" (4).

It is claimed that radionics can be used for the diagnosis of virtually all common diseases, for the detection of allergies, and also for the identification of individual bacterial pathogens. It is reported that radionics can cure such diverse conditions as asthma, schizophrenia, Hodgkin's disease, and coeliac disease. Further, it is said that radionics can correct breech presentation in pregnancy and even prevent dental caries.

### URINE THERAPY

In urine therapy, the patient's own urine is given orally, sublingually, intramuscularly or topically. A recent resurgence of urine therapy is associated with the notion that a therapeutic dose of specific antigen is excrete in the urine. This is associated with the concept of "auto-immune urine therapy" (5), and it is claimed that the method eliminates the need to identify specific allergens. It is asserted that urine therapy can cure gangrene, cancer, leukaemia, acute pyelonephritis, cardiac disease, malaria, orchitis, veneral disease, burns, nocturnal enuresis, obesity, psoriasis, asthma, glaucoma, arthritis, the common cold and Raynaud's phenomenon (7). It has also been claimed that injections of urine may have a beneficial effect on infectious hepatitis, asthma, hay-fever, urticaria, and migraine. The doses of urine for injection range from 2 mls for an infant to 8 mls for an adult.

Further interest in urine therapy stems from the notion that allergy to tap water is a common phenomenon, and that urine contains a substance which protects against allergy if a few drops of urine are administered sublingually. It is also claimed that sublingual urine therapy protects against allergy to members of one's family, a disorder which can allegedly be detected by the "hand-shaking test", in which allergy is confirmed if symptoms appear after two members of a family have held hands for 10 minutes (7).

### THE CYTOTOXIC TEST

Also known as the leucocyte cytoxicity test or the leucocyte food allergy test, this comprises the observation of morphological changes in white blood cells, incubated simultaneously with the appropriate antigen and the patient's serum. The presence or absence and degree of damage caused to the leucocytes is claimed to be an indicator of the presence of food and/or chemical sensitivity and to give some indication as to its degree.

### PULSE TESTING

Coca classified allergic disease into four main categories, but in addition he proposed a fifth group, familial non-reaginic food-allergy, which he named "idioblapsis". The features of this proposed condition were that individual

Correspondence to: Dr. T. J. David, Booth Hall Children's Hospital, Charlestown Road, Blackley, Manchester M9 2AA.

sensitivities were not attributable to circulating antibodies but to activity of the sympathetic nervous system, and that the allergic reaction reliably caused an acceleration of the pulse rate (8). Coca claimed that at least 90 per cent of the population suffered from idioblaptic allergy. He said that an increase in the pulse rate was a reliable pointer to food allergy and he recommended identification of idioblaptic allergy by observing a tachycardia five to 90 minutes after exposure to a food or inhaled material. Whilst acknowledging that other factors such as a fever or fear could cause an acceleration of the pulse, he claimed that subjects with idioblaptic allergy often wrongly ascribe their tachycardia to physical exercise (eg walking upstairs). He cited as an example a subject whose pulse rate only increased by two beats per minute after walking up two flights of stairs, whereas when the subject was suffering from an allergic headache "the mere rising from his seat" caused an increase of 10 to 15 beats per minute. Coca described the interpretation of the pulse as an "art" because of multiple confounding factors, including a latent period of lost sensitivity, and "aluminium sensitivity". Other authorities have suggested that a slowing of the pulse is also reliable. Using pulse testing, Coca claimed cures for headaches, urticaria, eczema, indigestion, colitis, haemorrhoids, hypertension, dysmenorrhoea, subfertility, frigidity and glaucoma. He reported that idioblaptic allergy reduced resistence to the common cold and to cancer of the breast, and he claimed that avoidance of pulse accelerating foods caused improvement of multiple sclerosis.

### OTHER TESTS EMPLOYING THE PULSE RATE

The so-called auricular cardiac reflex (a term originating in auricular medicine, a form of ear acupuncture) has been adapted for use by clinical ecologists. This reflex is said to be "a small movement of the position where the wrist pulse is strongest, either in the direction of the elbow or in the direction of the wrist" (9). A current text book of clinical ecology claims that if a dried food is brought near to the body, within half an inch of the skin but not actually touching the skin, then the auricular cardiac reflex changes and a diagnosis of food allergy can be made (9). Such testing currently employs dried food samples mounted in specially prepared filters, and it is claimed that with this technique 50 foods or chemicals can be tested in 15 minutes.

### VEGA ELECTRICAL TESTING FOR ALLERGY

Vega testing employs a Vegatest device. The method "relies on changes in the resistance to the flow of electricity over acupuncture points on the ends of fingers and toes, brought about by bringing particular substances, in glass phials, into series in the circuit" (10). Vega testing is used in clinical ecology to test for food or chemical sensitivity, and also to detect "organ stress", and it is reported that Vega testing is partially dependant on the "psyche" of the practitioner, to the extent that "changes in readings noted are partially psychokinetic effects (literally "mind-caused" effects), which may in some cases be observed extra-sensorily" (10).

### APPLIED KINESIOLOGY

The theory of applied kinesiology is that antigenic substances, if held near to the body, will cause a drop in the power of certain muscles. Where a patient is too young to co-operate with the testing, a surrogate is employed. The surrogate is tested alone, and then re-tested when holding the patient's hand.

### ENZYME POTENTIATED TRANSEPIDERMAL DESENSITIZATION

This technique is claimed to exploit a potentiating effect of betaglucuronidase when it is added to dilute allergen mixtures. A small plastic cup containing the appropriate desensitizing fluid is placed over an area of skin which has previously been scarified using a blunt scalpel. The cup is left in place for 24 hours. A mixture of more than 70 allergens can be applied in the hope that important ones have been included. Identification of all the patient's sensitivities is not necessarily required. Treatment is given monthly for three months with booster doses every four months.

### INTESTINAL CANDIDIASIS AND CANDIDA SENSITIVITY

Numerous symptoms have been attributed by ecologists to "intestinal candidiasis" and "dysbiosis". It is further claimed that schizophrenia, hypoglycaemia, myasthenia gravis, Crohn's disease, systemic lupus erythematosis, rheumatoid arthritis, psoriasis and multiple sclerosis are "yeast-related diseases" and that anti-yeast therapy results in recovery. There is disagreement amongst ecologists as to whether intestinal candidiasis should be treated by conventional anti-fungal therapy with nystatin or by sublingual or subcutaneous neutralization. It has been suggested that intestinal cadidiasis can underlie food sensitivity and that such food sensitivities will not respond to a diet or desensitization until the candidiasis itself has been diagnosed and treated.

### HAIR ANALYSIS

It is a belief of clinical ecology that abnormalities in trace metal status may "underpin" the development of food and chemical sensitivities. Hair trace metal analysis is claimed to be a useful investigation, to be accompanied by correction of any imbalance by a mineral supplement.

### INTRADERMAL SKIN TESTING

Conventional intradermal testing for food allergy produces numerous false positive results which are difficult to interpret. Conventional practitioners and ecologists agree it is not a helpful technique, and it carries a small risk of anaphylaxis.

However in a bizarre alternative technique, allergens are diluted with benzyl alcohol and normal saline, to produce nine successively weaker concentrations, and these are injected intradermally (10). A resulting weal is observed, and after 10 minutes assessed for features such as lateral increase in size, blanching, hardness and well demarcated edges. A "positive" weal is said to have retained most of these features and to have grown at least two mm in diameter (10). It is reported that using this method an injection of a concentrate may produce no reaction, whereas injection of a diluted solution may result in a positive weal. Further it is claimed that at the same time as the weal enlarges, symptoms related to the patient's complaint are provoked in 70% of subjects (10). Finally, it is said that if increasing dilutions are injected at half-hourly intervals, when a dilution is reached which causes no weal the injection of this is accompanied by an almost instantaneous cessation of symptoms (10). This dilution is then employed in neutralization therapy (see below). As with radionic testing it is reported that the mere process of intradermal testing is itself therapeutic, and it has been postulated that intradermal testing could alter the immune system "in a favourable direction".

### ORAL OR SUBCUTANEOUS NEUTRALIZATION

Having determined the "neutralizing dose" with the form of intradermal testing described above, neutralizing solutions are then administered either by sublingual drops or by subcutaneous injection. Sublingual drops are said to be beneficial for five hours, while injections are claimed to last at least two days. It is suggested that after a few weeks the injectios can be given every three days, and it is claimed that symptom control occurs almost immediately, though "permanent desensitization often requires a few years of therapy". Acknowledged drawbacks to the method include the patient who "constantly develops new allergies", and also "gradual changes in the body's response which involve a parallel change in the concentration of the drops to neutralize the allergens". For treatment, neutralizing doses of all the antigenic items are combined in a single solution and either injected subcutaneously or given sublingually.

Most patients seen in recent years in our department who have been previously treated with sublingual neutralization drops have been diagnosed as suffering not only from food allergy, but also from allergy to car exhaust fumes and North Sea gas. It is not clear how the diagnosis of all these allergies has been established. However, the ecological view seems to be that allergy to North Sea gas is so ubiquitous that diagnostic testing is unnecessary.

### ABSENCE OF OBJECTIVE VALIDATION OF THESE METHODS

It is held that radionic techniques are not amenable to any sort of scientific study, and that radionics "does not have a place within such a restricted belief system as orthodox science. Radionics is an interface with higher dimensions of reality and consciousness, where the gods of logic and reason do not necessarily hold sway" (11). Scientific study is reportedly precluded by the fact that disbelief either in the patient's mind or that of a third party destroys the reliability of the testing process. "Psionic medicine requires of the practitioner a mind undisturbed by the clash of disputation—a state not easy to obtain in the presence of those whose coin is dispute" (4). The use of urine therapy is based on a few poorly documented anecdotes, and there appear to be no scientific or objective studies of its use. Cytotoxic testing has been shown to be unreliable, in that it fails to diagnose known food allergy, it produces a large number of false positive results, there is poor correlation between observers reading the same test blind, and there is fluctuation of results in individual subjects from day to day and from week to week. There do not appear to have been any published attempts to objectively verify pulse testing, Vega testing, applied kinesiology, enzyme potentiated desensitisation, dysbiosis or intestinal candidiasis as a cause of allergy. There is no objective evidence that mineral supplementation, based on the results of hair trace metal analysis, confers any clinical benefit in the field of allergy, and it has been clearly shown that hair trace metal analysis is technically unreliable and of no value in an individual patient (12). Attempts to validate the kind of intradermal testing described above have been unsuccessful. There appear to have been no attempts to objectively validate the diagnosis of multiple food allergy combined with allergy to car exhaust fumes and North Sea Gas, or the treatment of these supposed entities with sublingual drops.

### SEQUELAE OF BOGUS ALLERGY TREATMENT

Local experience has been that the use of the above methods is associated with serious sequelae. Many of these methods were associated with the false diagnosis of allergy or the creation of fictitious disease entities such as North Sea Gas allergy or dysbiosis. The application of these techniques is often associated with a failure to recognise and treat genuine disease (1). In many instances these methods have led to inappropriate and nutrionally inadequate diets (1), employed without the help of a dietician, whose supervision is particularly important where a child is to be treated with an elimination diet. In several cases bogus allergy diagnosis has caused an increasing obsession with allergy as a cause of disease, leading to a treadmill of progressively more bizarre dietary restrictions. Finally, visits to ecology, environmental therapy or private allergy clinics have resulted in serious financial hardship. In a consecutive series of 35 patients we have seen who previously received bogus allergy treatment outside the National Health Service the mean cost was £159, ranging from £6.00 to £800, and in 11 patients the cost was £200 or more.

### CONCLUSIONS

There must be concern about doctors who without any specialist training are setting up allergy, ecology or environmental therapy clinics. Bogus methods, cloaked in the apparatus of science, are associated with dishonest and false claims for cure, the diagnosis of non-existent disease, dangerous and inappropriate diets, failure to diagnose and treat important and treatable disease, and serious financial hardship incurred by hard-up families desperate for a cure. The overenthusiastic, uncritical and inappropriate application of allergy treatment may be detrimental and costly, and it is increasing.

### REFERENCES

1. DAVID, T. J. (1985) The overworked or fraudulent diagnosis of food allergy and food intolerance in children. J. Roy. Soc. Med. supp. 5, **78**, pp. 21–31.
2. FULDER, S. (1984) The Handbook of Complementary Medicine. London, Hodder & Stoughton.
3. REYNER, J. H., LAURENCE, G., UPTON, C. (1982) Psionic Medicine. 2nd ed. London, Routledge & Kegan Paul.
4. LOCKER, L. (1983) Holistic Healing for Dowsers. Bristol, Lithprint.
5. WILSON, C. W. M., LEWIS, A. (1983) Auto-immune urine therapy against human allergic disease: a physiological self defence factor. Med. Hypotheses. **12**, pp. 143–158.
6. ARMSTRONG, J. (1981) The Water of Life. A Treatise on Urine Therapy. 2nd ed. Saffron Walden, Health Science Press.
7. WILSON, C. M. W. (1983) Allergic factors affecting the family. Nutrition & Health **1**, pp. 195–207.
8. COCA, A. F. (1953) Familial Nonreaginic Food-Allergy. 3rd ed. Illinois, Charles C. Thomas.
9. LEWITH, G. T., KENYON, J. N. (1985) Clinical Ecology. Wellingborough, Thorsons.
10. KENYON, J. N. (1986) 21st Century Medicine. A Layman's Guide to the Medicine of the Future. Wellingborough, Thorsons.
11. TANSLEY, D. V. (1982) Radionics: Science or Magic? An Holistic Paradigm of Radionic Theory and Practice. Saffron Walden, C. W. Daniel.
12. TAYLOR, A. (1986) Usefulness of measurements of trace elements in hair. Ann. Clin. Biochem. **23**, pp. 364–378.

